# Einsamkeit in Deutschland – Prävalenz, Entwicklung über die Zeit und regionale Unterschiede

**DOI:** 10.1007/s00103-024-03937-y

**Published:** 2024-08-22

**Authors:** Theresa M. Entringer, Barbara Stacherl

**Affiliations:** 1 Sozio-oekonomisches Panel (SOEP), Deutsches Institut für Wirtschaftsforschung e. V. (DIW Berlin), Mohrenstraße 58, 10117 Berlin, Deutschland; 2 Institut für Psychologie, Universität Greifswald, Greifswald, Deutschland

**Keywords:** Einsamkeit, Einsamkeitsprävalenzen, Veränderungen in Einsamkeit, Regionale Unterschiede, Covid-19-Pandemie, Loneliness, Loneliness prevalences, Changes in loneliness, Regional differences, COVID-19 pandemic

## Abstract

**Hintergrund:**

Einsamkeit ist weitverbreitet und hat negative Folgen für die Gesundheit. Diese Studie soll die Fragen beantworten: (1) wie viele Menschen in Deutschland hocheinsam sind, (2) wie sich die Einsamkeit in Deutschland über die Zeit – insbesondere im Hinblick auf die Covid-19-Pandemie – veränderte und (3) welche regionalen Unterschiede es in der Einsamkeit in Deutschland gibt.

**Methoden:**

Die vorliegende Studie verwendet Daten aus den Wellen 2013, 2017 und 2021 des Sozio-oekonomischen Panels, einer deutschen bevölkerungsrepräsentativen Panelstudie. Auf der Grundlage der University of California, Los Angeles(UCLA)-Einsamkeitsskala schätzen wir die Prävalenzen der Hocheinsamen. Mit Mehrebenenmodellen analysieren wir die Veränderungen der Einsamkeit von 2013 bis 2021. Schließlich erstellen wir Karten, um die regionale Verteilung der Einsamkeit in Deutschland zu illustrieren.

**Ergebnisse:**

Rund 2 % der in Deutschland lebenden Menschen gehören zu den hocheinsamen Menschen. Dieser Anteil änderte sich auch während der Covid-19-Pandemie nicht. Allerdings stieg die mittlere Einsamkeit in Deutschland an, vor allem während der Pandemie. Gleichzeitig ändert sich auch die regionale Verteilung von Einsamkeit. Während 2013 vor allem der Osten Deutschlands von Einsamkeit betroffen war, gehören während der Pandemie vor allem Regionen im Westen Niedersachsens, Rheinland-Pfalz und Hessen zu den am stärksten von Einsamkeit betroffenen Regionen.

**Diskussion:**

Ursachen für diese Veränderungen können sowohl in strukturellen Veränderungen der deutschen Gesellschaft in den vergangenen 10 Jahren liegen, aber auch an unterschiedlichen Maßnahmen zur Bekämpfung der Covid-19-Pandemie. Da die neuesten Daten zu Einsamkeit aus 2021 stammen, bedarf es dringend weiterer Datenerhebungen, um die aktuelle Einsamkeit in Deutschland abzubilden.

## Abstract (en)

**Background:**

Loneliness is widespread and has negative health consequences. The present study seeks to answer the following questions: (1) how many people in Germany are highly lonely, (2) how has loneliness changed in Germany over time—in particular with regard to the COVID-19 pandemic, and (3) how is loneliness distributed regionally in Germany today?

**Methods:**

The present study uses data from the 2013, 2017, and 2021 waves of the Socio-Economic Panel, a population representative panel study from Germany. Based on the University of California, Los Angeles (UCLA) loneliness scale, we estimate prevalences for high loneliness for different risk groups in Germany. Further, using multi-level models, we estimate the changes in loneliness from 2013 to 2021. Finally, we generate regional maps to display the relative regional distribution of loneliness in Germany over the three assessment waves.

**Results:**

The results show that around 2% of all people living in Germany are highly lonely, and this proportion has not changed during the COVID-19 pandemic. However, mean overall loneliness in Germany increased in the last decade, particularly during the pandemic. Additionally, the regional distribution of loneliness also changed. While eastern Germany was particularly affected by loneliness in 2013, western Lower Saxony, Rhineland-Palatine, and Hesse were the most affected regions during the pandemic.

**Discussion:**

Reasons for these changes could be structural changes in the German society over the past 10 years or the COVID-19 pandemic and the different measures implemented to combat the spread of the COVID-19 virus. However, since the latest representative data on loneliness stems from 2021, further data collection is urgently needed to depict the current state of loneliness in Germany.

## Hintergrund

Einsamkeit entsteht, wenn Menschen das Gefühl haben, dass ihre vorhandenen sozialen Beziehungen nicht ihren Bedürfnissen entsprechen [1]. Dies kann sowohl bedeuten, dass sich Menschen *mehr* soziale Beziehungen wünschen, als sie haben, aber auch, dass sie sich *tiefere *und *bessere* Beziehungen wünschen, als sie aktuell haben. Einsamkeit ist ein subjektives Gefühl, das aus der Wahrnehmung einer Diskrepanz zwischen erlebter Realität und Bedürfnis entsteht, und unterscheidet sich daher von objektiven Maßen wie sozialer Isolation oder Kontakthäufigkeit [2].

Die Gesundheitsforschung hat sich erst in den letzten 2 Jahrzehnten zunehmend mit Einsamkeit und deren Folgen für die Gesundheit auseinandergesetzt. Trotz dieses relativ jungen Forschungsfeldes ist heute anerkannt, dass Einsamkeit zwar keine Krankheit per se ist, jedoch ein erhebliches Gesundheitsrisiko darstellt [2, 3]. Beispielsweise geht Einsamkeit mit einem erhöhten Risiko für kardiovaskuläre Erkrankungen, Schlaganfälle und Typ-2-Diabetes einher [4, 5] und auch das Risiko für psychische Erkrankungen wie Depression, Angststörungen oder Suchterkrankungen ist erhöht [6]. Insgesamt konnte gezeigt werden, dass chronische Einsamkeit mit erhöhter Sterblichkeit und einer verkürzten Lebenserwartung zusammenhängt [3, 7].

Aufgrund dieser erheblichen gesundheitlichen Folgen hat die Bundesregierung im Dezember 2023 eine Strategie gegen Einsamkeit verabschiedet, deren erklärtes Ziel es ist, über Einsamkeit aufzuklären und für dieses Thema zu sensibilisieren [8]. Gleichzeitig wird in diesem Strategiepapier zu mehr Forschung zu Einsamkeit aufgerufen. Das wachsende Forschungsfeld zu Einsamkeit hat, international und in Deutschland, bereits eine Vielzahl von Risikofaktoren für Einsamkeit [5, 9–11] und Folgen von Einsamkeit identifiziert [3, 4]. Fragen zur Prävalenz von Einsamkeit, zur Entwicklung über die Zeit, insbesondere mit Hinblick auf die Covid-19-Pandemie, und zu regionalen Unterschieden sind allerdings weiter nicht ausreichend beforscht.

Zu Prävalenzen von Einsamkeit ist noch wenig bekannt, weil die vorliegenden Studien in der Regel nicht auf bevölkerungsrepräsentativen Daten basieren und daher keine Prävalenz von Einsamkeit schätzen können. Eine der wenigen Prävalenzschätzungen, die bisher zu Einsamkeit in Deutschland vorliegt, stammt von der Erstautorin der vorliegenden Studie und bezieht sich auf die Zeit vor und während der Hochphasen der Covid-19-Pandemie [12]. Darin werden Aussagen über von Einsamkeit bedrohte Menschen getroffen, d. h. über Menschen, die angeben, sich *zumindest manchmal* einsam zu fühlen. Diese Studie schätzte, dass die Prävalenz der von Einsamkeit bedrohten Menschen vor der Pandemie bei ca. 14 % und während der Pandemie im Jahr 2020 und zu Beginn des Jahres 2021 bei ca. 40 % lag. Wissen über die von Einsamkeit bedrohten Menschen ist hochrelevant für die Entwicklung zielgruppenspezifischer Präventionsmaßnahmen, es fehlen dabei aber Zahlen zu den *hocheinsamen* Menschen, also zu Menschen, die angeben, sich sehr oft oder immer einsam zu fühlen, und für die Einsamkeit daher ein besonders großes Gesundheitsrisiko darstellt [3, 13].

Außerdem ist bisher nicht ausreichend geklärt, wie sich die Einsamkeit über die Zeit und vor allem in Hinblick auf die Covid-19-Pandemie verändert hat. National und international gibt es nur wenig Evidenz für einen signifikanten Anstieg der Einsamkeit in der Zeit vor der Pandemie [14, 15]. Für die Zeit der Pandemie ist ein Anstieg der Einsamkeit etwas besser belegt [16–18]. Diese Studien basieren allerdings auf Daten aus der Hochphase der Pandemie und nur wenige Studien stammen aus Deutschland. Internationale Studien sind jedoch für die Situation in Deutschland aufgrund der großen Unterschiede in der Strenge der Kontaktrestriktionen zur Bekämpfung der Pandemie wenig aussagekräftig. Damit ist es bisher unklar, wie es den Menschen in Deutschland nach den Kontaktrestriktionen hinsichtlich ihrer Einsamkeit ging.

Schließlich sind regionale Unterschiede in der Einsamkeit bisher wenig erforscht. Einige Studien konnten zeigen, dass das individuelle Einsamkeitsgefühl nicht nur mit soziodemografischen Faktoren oder Persönlichkeitseigenschaften zusammenhängt, sondern auch regional variiert [19, 20]. Eine Studie auf Basis der Daten des Sozio-oekonomischen Panels (SOEP) aus dem Jahr 2013 fand, dass vor allem Veränderungen in der Bevölkerungszusammensetzung eine wichtige Rolle für Einsamkeit spielten. (Binnen‑)Migration, Veränderungen in den Wirtschaftsstrukturen und der politischen Landschaft in Deutschland haben im letzten Jahrzehnt die Bevölkerungszusammensetzung in den einzelnen Regionen in vielerlei Hinsicht weiter verändert [21–23]. Aus diesem Grund scheint es naheliegend, dass auch die regionale Verteilung von Einsamkeit in den letzten Jahren nicht stabil blieb. Bisher gibt es jedoch keine Studie, die die Frage beantwortet, wie sich die regionale Einsamkeit seit 2013 veränderte und vor allem in welchen Regionen in Deutschland die Menschen heute besonders einsam sind.

Auf Basis aktueller Daten des SOEP will die vorliegende Studie daher folgende 3 offene Fragen beantworten: Wie viele Menschen in Deutschland gehören zu den hocheinsamen Menschen? Wie hat sich die Einsamkeit in Deutschland über die Zeit – insbesondere im Hinblick auf die Covid-19-Pandemie – verändert? Welche regionalen Unterschiede gibt es in der Einsamkeit in Deutschland?

## Methoden

### Datengrundlage

Die Datengrundlage für diese Studie bildet das SOEP, eine repräsentative, jährliche Befragung privater Haushalte in Deutschland, die seit 1984 stattfindet [24]. Das SOEP enthält neben Informationen zu soziodemografischen Merkmalen und Wohnort der Befragten auch Fragen zu Einsamkeit. Einsamkeit wurde im SOEP bisher in 3 Wellen bei erwachsenen Befragten (ab 18 Jahren) erhoben, das erste Mal im Jahr 2013, dann im Jahr 2017 und erneut 2021. Diese 3 Erhebungswellen liegen den Ergebnissen in diesem Artikel zugrunde. Hinsichtlich der Daten aus 2021 sei an dieser Stelle angemerkt, dass es sich hierbei um die Daten aus der SOEP-Kernbefragung handelt, die von Mai 2021 bis Januar 2022 erhoben wurden, also zu einer Zeit, als es bereits einen Impfstoff gab und davon auszugehen war, dass keine weiteren flächendeckenden Lockdowns eintreten würden. Damit lassen die unten stehenden Ergebnisse einen ersten Ausblick auf die Zeit nach den coronabedingten Kontaktrestriktionen zu.

### Messinstrumente

#### Messung von Einsamkeit.

Das SOEP misst Einsamkeit mithilfe der etablierten UCLA-Einsamkeitsskala [25]. Dieser Fragebogen umfasst die folgenden 3 Fragen:

1. Wie oft haben Sie das Gefühl, dass Ihnen die Gesellschaft anderer fehlt?
2. Wie oft haben Sie das Gefühl, außen vor zu sein?
3. Wie oft haben Sie das Gefühl, dass Sie sozial isoliert sind?

Innerhalb des SOEP-Interviews werden die Befragten gebeten, diese 3 Fragen mithilfe einer 5‑stufigen Antwortskala von „sehr oft“ bis „nie“ (1 = sehr oft, 2 = oft, 3 = manchmal, 4 = selten, 5 = nie) zu beantworten. Für die folgenden Analysen wurden aus den Antworten auf die 3 Fragen 2 unterschiedliche Einsamkeitsindizes gebildet. Erstens, um *hocheinsame* Personen zu identifizieren, wurde ein binärer Einsamkeitsindikator gebildet, auf dem Personen als hocheinsam klassifiziert wurden, die alle 3 Fragen mit sehr oft (1) oder oft (2) beantworteten. Zweitens, um die Veränderungen der Einsamkeit über die Zeit statistisch zu testen und regionale Unterschiede von Einsamkeit darzustellen, wurde die *mittlere* Einsamkeit für jede Person als Mittelwert der 3 Items berechnet. Für die Berechnung der mittleren Einsamkeit wurden die Items zunächst umgekehrt kodiert, sodass höhere Werte einer höheren Einsamkeit entsprechen.

#### Identifizierung von Risikogruppen.

Um Risikogruppen für hocheinsame Personen zu identifizieren, wurden 7 soziodemografische Merkmale mit in die Analysen einbezogen. Diese sind an der bestehenden Literatur zu Risikofaktoren für Einsamkeit orientiert [5, 9, 10].

1. *Alter:* Gemäß der Organisation für Economic Co-Operation and Development (OECD) Klassifizierung wurden 5 Altersgruppen betrachtet: (1) die 18- bis 30-Jährigen, (2) die 31- bis 45-Jährigen, (3) die 46- bis 60-Jährigen, (4) die 61- bis 75-Jährigen und (5) die über 75-Jährigen.
2. *Geschlecht:* Geschlecht wurde binär (Männer und Frauen) definiert.
3. *Einkommen:* Einkommensunterschiede wurden auf Basis des verfügbaren Haushaltseinkommens analysiert. Dieses umfasst alle Einkunftsarten auf Haushaltsebene abzüglich Steuern auf Einkommen und Sozialversicherungsabgaben und zuzüglicher staatlicher Transferleistungen. Das Haushaltseinkommen wurde mit der sogenannten Quadrat-Wurzel-Äquivalenzskala gemäß der Haushaltsgröße bedarfsangepasst. Das Haushaltseinkommen wurde zudem mit dem Verbraucherpreisindex angepasst. Aus der Verteilung dieses Einkommens wurden 3 gleich große Gruppen – Terzile – gebildet, die den oberen, mittleren und unteren materiellen Lebensstandard in Deutschland widerspiegeln.
4. *Direkter Migrationshintergrund:* In den Analysen wurde unterschieden zwischen Personen mit direktem Migrationshintergrund und Personen ohne direkten Migrationshintergrund. Dabei hat eine Person einen direkten Migrationshintergrund, wenn sie nicht in Deutschland geboren wurde.
5. *Haushaltsform:* Hinsichtlich der Haushaltsform wurde zwischen alleinlebenden Personen und Personen, die mit anderen Menschen gemeinsam in einem Haushalt leben, unterschieden.
6. *Kinder:* In den Analysen wurde unterschieden zwischen Personen mit Kind(ern) und kinderlosen Personen.
7. *Regionale Unterschiede:* Für die Prävalenzschätzungen wurde unterschieden zwischen Personen, die in Ostdeutschland leben, und Personen, die in Westdeutschland leben. Für die Analysen hinsichtlich der Veränderung von Einsamkeit über die Zeit sowie für die Darstellung der regionalen Unterschiede in der mittleren Einsamkeit wurde auf feingliedrigere regionale Indikatoren zurückgegriffen: Raumordnungsregionen und Gemeinden (für Details siehe statistische Analysen).

### Statistische Analysen

Die statistischen Analysen wurden in 3 Schritten durchgeführt. Zunächst wurden die Prävalenzen für hocheinsame Personen berechnet. Danach wurden die Veränderungen von Einsamkeit über die Zeit analysiert und schließlich regionale Unterschiede und deren Veränderungen über die Zeit grafisch dargestellt.

#### Prävalenzschätzungen.

Tab. 1 zeigt eine Übersicht der Stichprobengrößen in 2013, 2017 und 2021, die in die Prävalenzberechnung eingegangen sind. Die 3 Einsamkeitsitems wurden in den oben dargestellten Einsamkeitsindikator für hocheinsame Personen umkodiert. Die dazugehörigen Werte wurden in prozentualen Anteilen an der Gesamtbevölkerung ausgewiesen. Neben der Prävalenz in der Gesamtbevölkerung wurden gruppenspezifische Prävalenzen entlang der oben dargestellten soziodemografischen Charakteristika berechnet. Die Prävalenzberechnungen wurden außerdem getrennt für die 3 Einsamkeitsitems durchgeführt. Für alle Berechnungen wurde ein Gewichtungsverfahren gewählt, das Designeffekte und Non-Response berücksichtigt und damit die Verallgemeinerung auf die Gesamtpopulation der in Deutschland in privaten Haushalten lebenden erwachsenen Menschen zulässt [26].

**Tab. 1:** Übersicht über die den Prävalenzberechnungen zugrunde liegenden Stichprobengrößen in 2013, 2017 und 2021 auf Grundlage von Daten des Sozio-oekonomischen Panels (SOEP)

*Gruppierungsvariable*	Hocheinsame Personen			„Gefühl, dass Gesellschaft anderer fehlt“			„Gefühl, außen vor zu sein“			„Gefühl, sozial isoliert zu sein“		
	*2013*	*2017*	*2021*	*2013*	*2017*	*2021*	*2013*	*2017*	*2021*	*2013*	*2017*	*2021*
*Alter*												
18–30	14.645	15.213	9768	14.643	15.188	9495	14.624	15.116	9425	14.637	15.137	9807
31–45	1907	2141	1415	1909	2140	1412	1896	2129	2129	1907	2134	1419
46–60	4558	7570	4241	4558	7535	4114	4546	7494	4094	4555	7526	4257
61–75	9070	10.514	7534	9071	10.512	7461	9041	10.481	7417	9063	10.500	7525
Über 75	4907	4939	3844	4910	4939	3835	4889	4927	3806	4902	4933	3845
*Geschlecht*												
Männlich	15.828	18.809	12.991	15.829	18.773	12.780	15.792	18.702	12.696	15.822	18.748	12.995
Weiblich	19.259	21.568	13.803	1.262	21.541	13.529	19.204	21.445	13.429	19.242	21.482	13.850
*Einkommen*												
Niedrig	11.676	16.585	11.237	11.683	16.533	10.807	11.638	16.398	10.695	11.669	16.483	11.298
Mittel	11.256	10.996	5913	11.256	10.996	5911	11.235	10.980	5879	11.246	10.978	5914
Hoch	10.878	10.954	7747	10.875	10.953	7745	10.861	10.941	7731	10.874	10.942	7746
*Migrationshintergrund*												
Kein direkter	30.519	27.599	17.437	30.521	27.594	17.441	30.461	27.561	17.373	30.500	27.559	17.429
Direkter	4568	12.778	9365	4570	12.720	8876	4535	12.586	8760	4564	12.671	9424
*Alleinlebend*												
Alleinlebend	3603	4552	3775	3604	4549	3776	3593	4533	3743	3602	4540	3769
Mit anderen lebend	31.484	35.825	23.027	31.487	35.765	22.541	31.403	35.614	22.390	31.462	35.690	23.084
*Kinder*												
Kinderlos	29.182	31.352	20.033	29.184	31.303	19.628	29.107	31.170	19.481	29.167	31.240	20.092
Mindestens ein Kind	5905	9025	6769	5907	9011	6689	5889	8977	6652	5897	8990	6761
*Region*												
Westen	27.632	32.816	21.735	27.636	32.757	21.293	27.554	32.601	21.124	27.615	32.679	21.776
Osten	7455	7561	5067	7455	7557	5024	7442	7546	5009	7449	7551	5077
*Gesamt*	*35.087*	*40.377*	*26.802*	*35.091*	*40.314*	*26.317*	*34.996*	*40.147*	*26.133*	*35.064*	*40.230*	*26.853*

Hinweis: Bei der Gruppierungsvariable „Einkommen“ ist die Summe der untersuchten Personen kleiner als die unter „Gesamt“ ausgewiesenen Personen, da es bei dieser Variable Missings gab

#### Veränderungen über die Zeit.

Für Analysen von Veränderungen über die Zeit wurde ein multivariates Regressionsmodell verwendet, in dem die mittlere Einsamkeit als abhängige Variable verwendet wurde und in das 2 binäre Variablen für die Erhebungsjahre 2017 und 2021 eingefügt wurden. Damit wird die Veränderung der Einsamkeit im Vergleich zum Basiserhebungsjahr 2013 dargestellt. Darüber hinaus wurde für die oben genannten soziodemografischen Merkmale kontrolliert. Um dem regionalen Clustering Rechnung zu tragen, wurde ein Mehrebenenmodell mit den 96 Raumordnungsregionen als Random Intercept (d. h. Ebene 2) verwendet.^1^ Das Modell wurde einmal für die mittlere Einsamkeit und je einmal einzeln mit den 3 invertierten Einsamkeitsitems berechnet.

#### Regionale Unterschiede.

Um regionale Muster von Einsamkeit darzustellen, wurden Einsamkeitskarten für Deutschland erstellt. Für die Erstellung der Karten wurde eine sogenannte Actor-Based-Clustering-Methode verwendet [19, 27]. Diese Methode erlaubt es, durch Schätzungen auf Ebene der Gemeinden Einsamkeit sehr kleinräumig abzubilden. Die Einsamkeitswerte auf Gemeindeebene werden hierbei mit einem Distanz-gewichteten Mittelwert geschätzt, der alle Befragten einbezieht und Personen in unmittelbarer Nähe höher gewichtet.^2^ Daraus wurden schließlich Einsamkeitskarten erstellt, die für jedes Jahr die *relative* Verteilung der Einsamkeit in Deutschland abbilden. Damit können Aussagen getroffen werden, welche Regionen stärker von Einsamkeit betroffen waren. Unterschiede sind als Standardabweichungen des jeweiligen Jahres angegeben. Darüber hinaus wurden Karten mit der *absoluten* Änderung der Einsamkeit erstellt. Damit können Aussagen getroffen werden, welche Regionen stärker von Änderungen der Einsamkeit betroffen waren.

## Ergebnisse

### Einsamkeitsprävalenzen

Tab. 2 zeigt die Anteile der hocheinsamen Personen über die Zeit, getrennt für verschiedene Personengruppen. Tab. 3 können die Anteile jener Personen entnommen werden, die die 3 Items zur Messung von Einsamkeit jeweils mit „oft“ oder „sehr oft“ beantwortet haben. In der untersten Zeile der Tabellen sind jeweils die Gesamtzahlen für die in Deutschland lebenden Menschen zu finden. In der untersten Zeile von Tab. 2 ist zu erkennen, dass sowohl im Jahr 2013 als auch im Jahr 2017 knapp 2 % aller in Deutschland lebenden Menschen hocheinsam waren (2013: 1,86 % und 2017: 1,99 %). Im Jahr 2021, gegen Ende der Covid-19-Pandemie, ist diese Zahl auf über 2 % gestiegen – die Prävalenz im Jahr 2021 unterscheidet sich jedoch, wie an den sich überschneidenden Konfidenzintervallen zu erkennen, nicht signifikant von den Vorjahreszahlen.

**Tab. 2:** Übersicht über die Anteile hocheinsamer erwachsener Personen ab 18 Jahren in Deutschland über die Zeit. Datengrundlage: SOEP

*Gruppierungsvariable*	Hocheinsame Personen (Anteil in %)		
	*2013*	*2017*	*2021*
*Alter*			
18–30	1,62 [1,30; 1,95]	1,89 [1,52; 2,26]	2,93 [2,24; 3,62]
31–45	2,74 [1,86; 3,62]	3,20 [2,19; 4,21]	1,62 [0,72; 2,53]
46–60	1,04 [0,67; 1,40]	2,06 [1,49; 2,64]	2,40 [1,50; 3,29]
61–75	2,41 [1,84; 2,97]	1,92 [1,48; 2,37]	2,28 [1,68; 2,89]
Über 75	1,54 [1,05; 2,03]	1,47 [0,98; 1,96]	1,70 [1,15; 2,25]
*Geschlecht*			
Männlich	1,43 [1,11; 1,74]	1,58 [1,28; 1,88]	1,89 [1,45; 2,33]
Weiblich	2,26 [1,92; 2,60]	2,39 [2,04; 2,74]	2,72 [2,25; 3,20]
*Einkommen*			
Niedrig	3,95 [3,30; 4,59]	3,67 [3,10; 4,25]	3,66 [2,98; 4,34]
Mittel	1,50 [1,15; 1,84]	1,51 [1,15; 1,87]	2,26 [1,63; 2,90]
Hoch	0,50 [0,31; 0,69]	0,67 [0,47; 0,86]	1,15 [0,75; 1,56]
*Migrationshintergrund*			
Kein direkter	1,67 [1,44; 1,90]	1,57 [1,35; 1,79]	2,03 [1,70; 2,36]
Direkter	3,63 [2,51; 4,75]	4,04 [3,23; 4,84]	3,73 [2,73; 4,73]
*Alleinlebend*			
Alleinlebend	3,41 [2,68; 4,14]	3,22 [2,52; 3,93]	3,22 [2,36; 4,08]
Mit anderen lebend	1,39 [1,18; 1,59]	1,63 [1,41; 1,84]	2,04 [1,71; 2,37]
*Kinder*			
Kinderlos	1,89 [1,63; 2,15]	2,12 [1,84; 2,40]	2,25 [1,88; 2,62]
Mindestens ein Kind	1,77 [1,28; 2,26]	1,68 [1,27; 2,09]	2,45 [1,81; 3,09]
*Region*			
Westen	1,74 [1,49; 1,99]	1,91 [1,66; 2,16]	2,17 [1,83; 2,52]
Osten	2,35 [1,75; 2,95]	2,39 [1,84; 2,94]	3,01 [2,13; 3,90]
*Gesamt*	*1,86 [1,62; 2,09]*	*1,99 [1,76; 2,22]*	*2,32 [1,99; 2,64]*

Hinweise: Die Prävalenzen basieren auf einem binären Einsamkeitsindikator, mit dem alle Personen als hocheinsam klassifiziert wurden, die alle 3 Fragen der University of California, Los Angeles (UCLA) Einsamkeitsskala mit sehr oft (1) oder oft (2) beantwortet haben. Die hier angegebenen Prävalenzen gelten für alle erwachsenen Personen ab 18 Jahren, die in Deutschland in privaten Haushalten leben. 95 %ige Konfidenzintervalle sind in Klammern angegeben

**Tab. 3:** Übersicht über die Anteile der erwachsenen Personen ab 18 Jahren in Deutschland, die die 3 Items der UCLA-Einsamkeitsskala mit „oft“ oder „sehr oft“ beantworteten. Datengrundlage: SOEP

*Gruppierungsvariable*	Gefühl, dass Gesellschaft anderer fehlt (Anteil in %)			Gefühl, außen vor zu sein (Anteil in %)			Gefühl, sozial isoliert zu sein (Anteil in %)		
	*2013*	*2017*	*2021*	*2013*	*2017*	*2021*	*2013*	*2017*	*2021*
*Alter*									
18–30	10,06 [9,19; 10,94]	9,78 [8,95; 10,60]	20,40 [18,73; 22,08]	6,15 [5,40; 6,90]	6,64 [5,90; 7,37]	7,77 [6,69; 8,84]	4,72 [4,09; 5,36]	4,94 [4,30; 5,58]	8,70 [7,55; 9,84]
31–45	15,75 [13,70; 17,80]	13,71 [11,76; 15,65]	23,30 [20,18; 26,43]	8,67 [7,10; 10,25]	7,61 [6,13; 9,10]	7,61 [6,13; 9,10]	6,08 [4,79; 7,37]	5,38 [4,11; 6,65]	5,42 [3,74; 7,11]
46–60	10,90 [9,31; 12,48]	10,20 [9,00; 11,40]	18,33 [16,03; 20,63]	5,75 [4,64; 6,87]	7,42 [6,38; 8,45]	9,80 [8,08; 11,53]	4,16 [3,26; 5,07]	6,66 [5,66; 7,65]	9,82 [8,15; 11,48]
61–75	10,00 [8,94; 11,06]	7,68 [6,86; 8,49]	20,23 [18,49; 21,98]	7,45 [6,53; 8,37]	6,24 [5,48; 7,00]	7,14 [6,01; 8,27]	6,19 [5,34; 7,04]	5,12 [4,44; 5,81]	7,18 [6,07; 8,29]
Über 75	8,57 [7,52; 9,62]	9,42 [8,16; 10,67]	19,97 [17,99; 21,96]	5,06 [4,27; 5,85]	5,80 [4,85; 6,75]	5,93 [4,84; 7,02]	4,21 [3,41; 5,00]	3,64 [2,90; 4,38]	5,54 [4,43; 6,64]
*Geschlecht*									
Männlich	8,75 [8,01; 9,48]	7,92 [7,29; 8,55]	17,83 [16,56; 19,09]	5,54 [4,94; 6,14]	5,68 [5,12; 6,25]	6,65 [5,83; 7,47]	4,24 [3,72; 4,75]	4,68 [4,16; 5,20]	6,44 [5,64; 7,24]
Weiblich	12,01 [11,24; 12,78]	11,27 [10,55; 11,99]	22,61 [21,32; 23,91]	7,37 [6,76; 7,98]	7,49 [6,90; 8,07]	8,31 [7,48; 9,14]	5,87 [5,31; 6,43]	5,45 [4,95; 5,94]	8,57 [7,73; 9,40]
*Einkommen*									
Niedrig	15,54 [14,41; 16,68]	13,82 [12,81; 14,84]	23,36 [21,70; 25,03]	10,93 [9,92; 11,93]	10,61 [9,69; 11,52]	10,94 [9,74; 12,14]	10,25 [9,29; 11,21]	9,89 [9,01; 10,77]	11,35 [10,18; 12,52]
Mittel	10,06 [9,14; 10,99]	9,12 [8,27; 9,96]	20,79 [19,03; 22,54]	5,45 [4,77; 6,13]	5,82 [5,15; 6,50]	6,92 [5,87; 7,97]	3,73 [3,15; 4,31]	3,49 [2,96; 4,02]	7,55 [6,40; 8,71]
Hoch	6,82 [6,03; 7,62]	6,06 [5,39; 6,72]	17,23 [15,81; 18,65]	3,72 [3,15; 4,30]	3,44 [2,92; 3,96]	5,08 [4,21; 5,96]	1,97 [1,53; 2,41]	1,85 [1,49; 2,21]	4,00 [3,26; 4,74]
*Migrationshintergrund*									
Kein direkter	9,95 [9,40; 10,50]	8,39 [7,89; 8,89]	19,16 [18,21; 20,11]	6,26 [5,81; 6,70]	5,97 [5,54; 6,40]	6,68 [6,08; 7,28]	4,86 [4,46; 5,25]	4,38 [4,01; 4,75]	6,78 [6,18; 7,38]
Direkter	15,07 [13,07; 17,07]	15,69 [14,24; 17,14]	25,89 [23,25; 28,52]	8,66 [7,10; 10,23]	9,71 [8,55; 10,86]	11,68 [9,88; 13,48]	7,23 [5,80; 8,66]	8,45 [7,37; 9,54]	11,20 [9,50; 12,91]
*Alleinleben*									
Alleinlebend	15,75 [14,28; 17,22]	13,32 [12,01; 14,64]	22,07 [20,06; 24,08]	10,44 [9,18; 11,70]	9,57 [8,41; 10,73]	9,13 [7,72; 10,55]	8,36 [7,23; 9,50]	7,09 [6,09; 8,09]	9,41 [7,99; 10,83]
Mit anderen	8,83 [8,30; 9,36]	8,54 [8,05; 9,03]	19,73 [18,72; 20,74]	5,29 [4,89; 5,69]	5,73 [5,33; 6,13]	7,00 [6,37; 7,63]	4,09 [3,73; 4,45]	4,47 [4,12; 4,83]	6,95 [6,34; 7,57]
*Kinder*									
Kinderlos	10,30 [9,71; 10,88]	9,80 [9,23; 10,37]	21,48 [20,38; 22,59]	6,05 [5,59; 6,50]	6,25 [5,80; 6,71]	6,98 [6,30; 7,65]	4,90 [4,49; 5,31]	4,92 [4,52; 5,33]	7,12 [6,46; 7,79]
Mindestens ein Kind	10,81 [9,61; 12,01]	9,24 [8,33; 10,15]	17,83 [16,25; 19,41]	7,68 [6,67; 8,70]	7,45 [6,60; 8,31]	8,56 [7,43; 9,68]	5,58 [4,70; 6,46]	5,42 [4,69; 6,15]	8,35 [7,23; 9,47]
*Region*									
Westen	10,02 [9,42; 10,61]	9,46 [8,93; 10,00]	20,91 [19,88; 21,93]	6,16 [5,69; 6,62]	6,50 [6,05; 6,95]	7,01 [6,38; 7,64]	4,78 [4,37; 5,20]	4,90 [4,51; 5,29]	7,35 [6,70; 7,99]
Osten	12,21 [11,01; 13,41]	10,46 [9,38; 11,55]	17,23 [15,43; 19,03]	7,89 [6,85; 8,94]	7,12 [6,18; 8,06]	9,86 [8,34; 11,38]	6,36 [5,41; 7,31]	5,91 [5,02; 6,80]	8,41 [7,11; 9,71]
*Gesamt*	*10,43* *[9,90; 10,97]*	*9,64* *[9,15; 10,12]*	*20,28* *[19,38; 21,19]*	*6,49* *[6,06; 6,91]*	*6,61* *[6,20; 7,02]*	*7,50* *[6,92; 8,08]*	*5,08* *[4,70; 5,46]*	*5,07* *[4,71; 5,43]*	*7,53* *[6,95; 8,10]*

Hinweise: Die Prävalenzen geben den Anteil der jeweiligen Bevölkerungsgruppe an, die das jeweilige Item mit entweder sehr oft (1) oder oft (2) beantwortet hat. Die hier angegebenen Prävalenzen gelten für alle erwachsenen Personen ab 18 Jahren, die in Deutschland in privaten Haushalten leben. 95 %ige Konfidenzintervalle sind in Klammern angegeben

Hinsichtlich der Gruppierungsvariablen Alter, Geschlecht, Einkommen, Migrationshintergrund, Haushaltsform, Kinder und Region zeigt sich: Menschen zwischen 31 und 45 Jahren gehören in den Vorpandemiejahren häufiger zur Gruppe der Hocheinsamen als Menschen zwischen 18 und 30 Jahren und Menschen über 75. Während der Covid-19-Pandemie waren vor allem Menschen zwischen 18 und 30 Jahren betroffen; hier stieg der Anteil der Hocheinsamen vergleichsweise stärker an. Daneben gehörten über alle Erhebungsjahre mehr Frauen zur Gruppe der Hocheinsamen. Der Anteil der hocheinsamen Frauen stieg während der Pandemie nicht stärker an als der Anteil der hocheinsamen Männer; hier waren also beide Geschlechter gleichermaßen betroffen. Personen mit niedrigem Einkommen gehörten deutlich häufiger zur Gruppe der Hocheinsamen als Personen mit mittlerem und hohem Einkommen. Gleichzeitig stiegen die Anteile der Hocheinsamen während der Pandemie vorwiegend innerhalb der Gruppen mit mittleren und hohen Einkommen an, während die Anteile der Hocheinsamen unter den Personen mit niedrigem Einkommen unverändert blieben. Personen mit direktem Migrationshintergrund waren über alle Erhebungsjahre rund doppelt so häufig hocheinsam wie Personen ohne direkten Migrationshintergrund. Während der Anteil der Hocheinsamen ohne direkten Migrationshintergrund in der Covid-19-Pandemie leicht anstieg, deutete sich bei Menschen mit direktem Migrationshintergrund im Jahr 2021 ein leichter Rückgang an. Ferner zeigte sich, dass alleinlebende Personen 2017 fast doppelt so häufig zu den hocheinsamen Personen gehören wie Personen, die mit anderen leben. Interessanterweise veränderte sich die Einsamkeit unter den Alleinlebenden während der Covid-19-Pandemie kaum – während die mit anderen Menschen lebenden Personen deutliche Anstiege in ihrer Einsamkeit verzeichneten. Darüber hinaus zeigt Tab. 1, dass Kinderlose und Personen mit Kindern sich kaum hinsichtlich ihrer Einsamkeitsprävalenzen unterschieden.^3^ Außerdem zeigt sich, dass sich Unterschiede in Einsamkeit zwischen Ost- und Westdeutschland über die Zeit zu verändern scheinen. Während im Jahr 2013 Menschen in Ostdeutschland noch häufiger zu den hocheinsamen Personen gehörten als in Westdeutschland, näherten sich diese Unterschiede im Jahr 2017 an, wurden im Jahr 2021 jedoch wieder größer.

### Veränderungen über die Zeit

Tab. 4 zeigt die Ergebnisse des Regressionsmodells, in welchem die Veränderungen in Einsamkeit über die 3 Erhebungsjahre modelliert wurden. Analog zu den bereits oben dargestellten Gruppenunterschieden hinsichtlich der Einsamkeitsprävalenz wird im Modell für die gleichen soziodemografischen Merkmale kontrolliert. Die Ergebnisse bezüglich der Gruppenunterschiede bleiben stabil, auch wenn für die jeweils anderen soziodemografischen Variablen kontrolliert wird.

**Tab. 4:** Ergebnisse aus dem hierarchischen Modell zur Schätzung der Veränderung von Einsamkeit über die Zeit. Datengrundlage: SOEP

	Mittlere Einsamkeit	Gefühl, dass Gesellschaft anderer fehlt	Gefühl, außen vor zu sein	Gefühl, sozial isoliert zu sein
*Prädiktor*	*Punktschätzer [95* *%iges KI]*	*Punktschätzer [95* *%iges KI]*	*Punktschätzer [95* *%iges KI]*	*Punktschätzer [95* *%iges KI]*
Konstante	*2,11* [2,08; 2,13]	*2,34* [2,30; 2,38]	*2,16* [2,13; 2,19]	*1,82* [1,79; 1,85]
Jahr 2017	*0,02* [0,01; 0,03]	−0,01 [−0,03; 0,01]	*0,02* [0,01; 0,04]	*0,05* [0,03; 0,06]
Jahr 2021	*0,20* [0,18; 0,21]	*0,37* [0,35; 0,39]	*0,06* [0,05; 0,08]	*0,17* [0,15; 0,19]
Alter: 31–45	0,01 [−0,01; 0,03]	0,02 [0,00; 0,05]	0,00 [−0,02; 0,03]	−0,01 [−0,03; 0,01]
Alter: 46–60	−0,02 [−0,04; 0,00]	*−0,04* [−0,06; −0,01]	−0,01 [−0,03; 0,02]	−0,01 [−0,03; 0,02]
Alter: 61–75	*−0,10* [−0,12; −0,07]	*−0,08* [−0,10; −0,05]	*−0,11* [−0,13; −0,08]	*−0,11* [−0,13; −0,08]
Alter: über 75	*−0,09* [−0,11; −0,06]	−0,03 [−0,06; 0,01]	*−0,13* [−0,17; −0,10]	*−0,11* [−0,14; −0,08]
Weibliches Geschlecht	*0,07* [0,06; 0,08]	*0,09* [0,07; 0,10]	*0,09* [0,07; 0,10]	*0,04* [0,03; 0,05]
Mittleres Einkommen	*−0,23* [−0,24; −0,21]	*−0,18* [−0,20; −0,16]	*−0,18* [−0,19; −0,16]	*−0,33* [−0,35; −0,32]
Hohes Einkommen	*−0,35* [−0,36; −0,33]	*−0,27* [−0,29; −0,26]	*−0,28* [−0,30; −0,26]	*−0,49* [−0,51; −0,48]
Direkter Migrationshintergrund	*0,07* [0,06; 0,08]	*0,09* [0,07; 0,10]	*0,04* [0,02; 0,05]	*0,10* [0,08; 0,11]
Mind. ein Kind	−0,01 [−0,03; 0,00]	*0,05* [0,03; 0,07]	*−0,05* [−0,07; −0,03]	*−0,04* [−0,06; −0,02]
Alleinlebend	*0,15* [0,13; 0,16]	*0,19* [0,17; 0,21]	*0,11* [0,09; 0,13]	*0,13* [0,11; 0,15]
Intraklassenkoeffizient	0,01	0,01	0,01	0,01
Anzahl der Clustervariablen	96	96	96	96
*N*	75.119	74.762	74.469	74.915
R^2^ marginal	0,06	0,06	0,03	0,07
R^2^ konditional	0,07	0,06	0,03	0,08

Hinweise: Kursiv gedruckte Punktschätzer sind signifikant mit *p* < 0,05. 95 %-KI: 95 %-Konfidenzintervall, gibt einen Bereich an, in dem der tatsächliche Punktschätzer mit 95 %iger Sicherheit liegt. Die Konstante zeigt die Schätzung des Einsamkeitsmittelwerts für Personen im Alter von 18 bis 30 Jahren, männlichen Geschlechts, die über ein niedriges Einkommen verfügen, keinen Migrationshintergrund oder Kinder haben und mit anderen gemeinsam in einem Haushalt leben im Jahr 2013. Die Prädiktoren zeigen für die jeweilige Merkmalsausprägung die geschätzte Abweichung des Einsamkeitswerts im Vergleich zu dieser Personengruppe

Die Modellkonstante beschreibt die mittlere Einsamkeit im Jahr 2013 für unter 30-jährige Männer, die zu der untersten Einkommensgruppe gehören, keinen direkten Migrationshintergrund und keine Kinder haben sowie mit anderen gemeinsam in einem Haushalt leben. Der Wert liegt bei *b* = 2,11 und läge damit auf der Einsamkeitsskala zwischen „selten“ und „manchmal“, jedoch deutlich näher an „selten“. Betrachtet man dann die Veränderungen im Jahr 2017, zeigt sich in der Gesamteinsamkeit ein kleiner, jedoch signifikanter Anstieg im Vergleich zum Jahr 2013, *b* = 0,02. Im Jahr 2021, nach Ende langanhaltender Covid-19-bedingter Kontaktrestriktionen, zeigt sich ein massiver Anstieg in der Gesamteinsamkeit. Der geschätzte Anstieg aus diesem Jahr ist mit *b* = 0,20 10-mal so groß wie der Anstieg von 2013 auf 2017. Ein Vergleich mit den Werten für die Einzelitems zeigt jedoch, dass dieser Anstieg – anders als im Vorerhebungsjahr – vornehmlich auf einem Anstieg der Werte für das Item „Gefühl, dass Gesellschaft anderer fehlt“, beruht (*b* = 0,37), gefolgt von einem Anstieg bei dem Item „Gefühl, sozial isoliert zu sein“ (*b* = 0,17). Der Anstieg der Werte für das Item „Gefühl, außen vor zu sein“, ist im Jahr 2021 zwar ebenfalls noch signifikant, aber im Vergleich deutlich kleiner (*b* = 0,06).

### Regionale Unterschiede

**Abb. 1:**
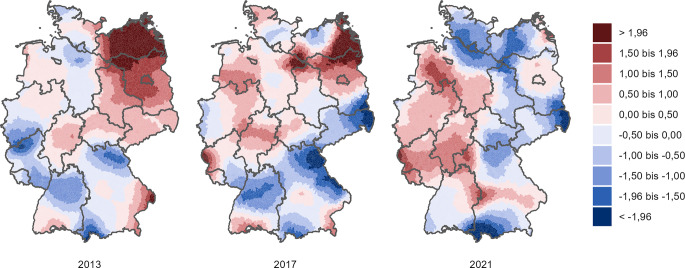
Regionale Unterschiede der Einsamkeit relativ zum Bundesdurchschnitt in den Jahren 2013, 2017 und 2021. Datengrundlage: Sozio-oekonomisches Panel (SOEP), Quelle: eigene Abbildung. *Hinweise: *Die Farben kodieren Standardabweichungen*, rot *steht für regionale Einsamkeit über dem Bundesdurchschnitt innerhalb des jeweiligen Erhebungsjahres,* blau *für regionale Einsamkeit darunter. Für die Interpretation der Karten sei hier angemerkt, dass die Farben relative regionale Unterschiede innerhalb eines Jahres angeben. Das bedeutet, dass sie keine Auskunft über die absolute Höhe des Einsamkeitswerts geben. Aus den Panels kann daher nicht geschlossen werden, dass *blaue* Regionen im Jahr 2021 weniger einsam waren als im Jahr 2013, sondern, dass *blaue* Regionen im Jahr 2021 weniger einsam waren als *rote* Regionen im Jahr 2021

Abb. 1 zeigt die Verteilung von Einsamkeit in Deutschland relativ zum Bundesdurchschnitt in den Jahren 2013, 2017 und 2021. Welche Regionen in einem Jahr jeweils am stärksten von Einsamkeit betroffen waren, war über die Zeit nicht konstant. Im Jahr 2013 waren Regionen in Mecklenburg-Vorpommern, Brandenburg und Berlin stärker von Einsamkeit betroffen, gefolgt von Regionen in Sachsen und Sachsen-Anhalt. Der Norden Baden-Württembergs, Rheinland-Pfalz und der Süden Nordrhein-Westfalens waren 2013 vergleichsweise weniger einsam. Die regionale Verteilung von Einsamkeit war im Jahr 2017 ähnlich zu 2013. Verglichen mit 2013 war jedoch der Einsamkeits-Hotspot in Mecklenburg-Vorpommern, Brandenburg und Berlin etwas kleiner und Sachsen und Bayern entwickelten sich eher zu Einsamkeits-Cold-Spots.

Im Jahr 2021 unterschied sich die regionale Verteilung von Einsamkeit schließlich vollständig von jener in 2013. In diesem Erhebungsjahr gehörten die Regionen in Ostdeutschland (mit Ausnahme von Brandenburg und Berlin) gemeinsam mit Schleswig-Holstein und der Bodenseeregion zu den Regionen mit der geringsten Einsamkeit in Deutschland. Regionen in Westdeutschland, insbesondere im westlichen Niedersachsen, in Nordrhein-Westfalen, Hessen und Rheinland-Pfalz, wiesen hingegen vergleichsweise mehr Einsamkeit auf.

Über die Zeit lässt sich also eine Verschiebung der von Einsamkeit stärker betroffenen Regionen von Ost- nach Westdeutschland feststellen. Hier ist anzumerken, dass dies für die mittlere Einsamkeit gilt, nicht aber für den Anteil der Hocheinsamen, der in beiden Landesteilen vergleichbar ist. Dies lässt sich durch die statistische Verteilung der Einsamkeit erklären, die in Ostdeutschland einen geringeren Mittelwert hat, aber breiter gestreut ist und damit mehr Menschen mit extremeren Einsamkeitswerten beinhaltet als in Westdeutschland.

**Abb. 2:**
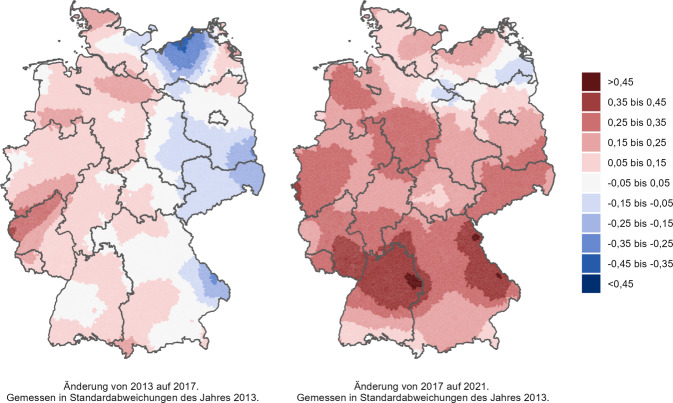
Veränderung der regionalen Einsamkeit in Deutschland von 2013 auf 2017 und von 2017 auf 2021. Datengrundlage: SOEP. Quelle: eigene Abbildung. Hinweise: Die Farben kodieren Änderungen der Einsamkeit,* rot *steht für einen Anstieg der regionalen Einsamkeit von einem Messzeitpunkt zum nächsten,* blau *für einen Rückgang der regionalen Einsamkeit von einem Messzeitpunkt zum nächsten, gemessen in Standardabweichungen des Jahres 2013

Neben der Betrachtung der Einsamkeitsmuster ist es aber auch wichtig, die Änderungen in der regionalen Einsamkeit mitzudenken (Abb. 2). Insgesamt war von 2013 auf 2017 nur ein kleiner Anstieg in der Einsamkeit deutschlandweit zu erkennen. Von 2017 auf 2021 stieg dann die Einsamkeit überall in Deutschland drastisch an. Die Umkehr der regionalen Verteilung von Einsamkeit kommt dadurch zustande, dass die Einsamkeit im Süden und Westen Deutschlands stärker anstieg als in Ostdeutschland.

## Diskussion

Die vorliegenden Analysen zeigen, dass sich der Anteil der *Hocheinsamen* trotz Covid-19-Pandemie in den letzten Jahren wenig verändert hat. Gleichzeitig stieg der Anteil der *von Einsamkeit bedrohten Personen* von 2013 bis 2021 und insbesondere während der Pandemie an [12]. Diese Ergebnisse lassen vermuten, dass die Pandemie zunächst keine schwerwiegenden Auswirkungen auf die Einsamkeit der in Deutschland lebenden Menschen hatte, wohl aber deutlich mehr Menschen sich „manchmal einsam“ fühlten.

Obwohl bereits belegt ist, dass höhere Einsamkeit mit stärkeren gesundheitlichen Risiken einhergeht [3, 13], sollten zukünftige Studien die unterschiedlichen Langfristfolgen für die beiden Einsamkeitsgruppen „Hocheinsame“ und „von Einsamkeit Bedrohte“ näher beleuchten. Die bereits an anderer Stelle dokumentierten Risikogruppen für Einsamkeit (z. B. Alleinlebende, Frauen; [11]) gelten teilweise auch für die hocheinsamen Menschen in Deutschland, vor allem in der Zeit vor der Covid-19-Pandemie. Während der Pandemie waren vor allem junge Menschen im Alter von 18 bis 30 Jahren betroffen, das bestätigt die bestehende Studienlage [17].

Während andere Studien Frauen und Menschen mit Migrationshintergrund als stärker von der Pandemie betroffene Gruppen identifizierten [12], zeigt sich in den vorliegenden Analysen ein differenzierteres Bild. Frauen und Menschen mit Migrationshintergrund waren sowohl vor als auch während der Covid-19-Pandemie deutlich stärker von Einsamkeit betroffen als Männer bzw. Menschen ohne Migrationshintergrund. Der Anstieg des Anteils der Hocheinsamen war 2021 bei Frauen und Männern allerdings ähnlich stark ausgeprägt und bzgl. Migrationshintergrund konnte nur bei Menschen ohne Migrationshintergrund ein Anstieg im Anteil der Hocheinsamen festgestellt werden.

Die weiteren Analysen belegen, dass die mittlere Einsamkeit über die 3 Erhebungsjahre signifikant anstieg; insbesondere war die mittlere Einsamkeit auch am Ende der Covid-19-Pandemie noch deutlich höher als in den Jahren zuvor. Der eher kleine Anstieg von 2013 auf das Jahr 2017 ist zum Teil durch den Zustrom Geflüchteter nach Deutschland zu erklären. Diese sind in der Regel viel stärker von Einsamkeit betroffen als Menschen in ihren Heimatländern [28]. Der Anstieg der mittleren Einsamkeit von 2017 auf 2021 ist vermutlich überwiegend mit der Covid-19-Pandemie zu erklären. Bereits andernorts wurden starke Anstiege während des ersten [16] und zweiten [29] Lockdowns verzeichnet. Die hier dargestellten Ergebnisse zeigen nun, dass auch nach Entwicklung des Impfstoffs, als die Kontaktrestriktionen im Wesentlichen beendet wurden, die mittlere Einsamkeit der in Deutschland lebenden Menschen noch deutlich höher war als vor der Pandemie. Der Anstieg der mittleren Einsamkeit ist dabei vor allem getrieben durch einen Anstieg im „Gefühl, dass die Gesellschaft anderer fehlt“. Dies lässt sich vermutlich auch damit erklären, dass die Menschen noch unter dem Eindruck des langen zweiten Lockdowns und den damit verbundenen Kontaktrestriktionen standen. Es scheint, dass durch die Kontaktrestriktionen nicht unmittelbar soziale Isolation hervorgerufen wurde, da Menschen per Telefon und Internet weiterhin in Kontakt bleiben konnten, aber dass direkte Nähe und gemeinsame Aktivitäten fehlten.

Die regionalen Analysen zeigen schließlich, dass sich die am stärksten von Einsamkeit betroffenen Regionen in Deutschland über die Zeit verändern. Während im Jahr 2013 vor allem die ostdeutschen Gebiete von Einsamkeit betroffen waren, sind es im Jahr 2021 vergleichsweise verstärkt die Gebiete in Westdeutschland. Mindestens 2 Gründe können bei diesen Entwicklungen eine Rolle gespielt haben: Zum einen kann die Binnenmigration auf unterschiedliche Weise mit regionalen Veränderungen der Einsamkeit zusammenhängen [19], beispielsweise können soziale Netzwerke durch den Zuzug oder Fortzug anderer beeinflusst werden, was wiederum Einflüsse auf die individuellen Einsamkeitsgefühle hat. Diese Idee wird ferner unterstützt durch wesentliche strukturelle Veränderungen im letzten Jahrzehnt [21–23], die zu verstärkter (Binnen‑)Migration in Deutschland geführt haben und dadurch die soziodemografische Zusammensetzung einzelner Regionen maßgeblich mitbeeinflussten. Zum anderen ist denkbar, dass auch die unterschiedlichen regionalen Kontaktrestriktionen während der Covid-19-Pandemie die Einsamkeit unterschiedlich beeinflusst haben. Beispielsweise lockerte die Landesregierung in Mecklenburg-Vorpommern deutlich vor Nordrhein-Westfalen ihre Kontaktrestriktionen [30], was Unterschiede in der regionalen Einsamkeit erklären könnte.

## Fazit

Die vorliegenden Analysen beruhen auf den einzigen bevölkerungsrepräsentativen Daten, die in Deutschland zur Einsamkeit von Menschen ab 18 Jahren vorliegen, und zeigen, dass auch gegen Ende der Covid-19-Pandemie ähnlich viele Menschen zu den Hocheinsamen gehörten wie vor Beginn der Pandemie. Gleichzeitig zeigen sich neue Risikogruppen (u. a. Menschen zwischen 18 und 30 Jahren) und auch eine andere regionale Verteilung von Einsamkeit als vor der Pandemie. Es bedarf dringend weiterer Datenerhebungen, um festzustellen, wie es aktuell, im Jahr 2024, um die Einsamkeit der in Deutschland lebenden Menschen steht. Besonders wünschenswert wären hierzu auch Einsamkeitsdaten zu den Personengruppen, die im SOEP nicht erhoben werden, beispielsweise jüngere und nicht in privaten Haushalten lebende Menschen.

## Footnotes

1 Um die Robustheit der Ergebnisse gegenüber anderen regionalen Clusterungen zu testen, wurden die Modelle außerdem einmal mit Bundesländern und einmal mit Kreisen als regionale Clusterung berechnet. Die Modelle führten zu konzeptuell identischen Ergebnissen.

2 Dafür wurde zunächst eine Distanzmatrix erstellt, die Distanzen zwischen allen Gemeinden beinhaltet. Daraus wurden mit einer Log-logistic-Funktion Distanzgewichte gebildet. Schließlich wurde für jede Gemeinde ein Distanz-gewichteter Mittelwert berechnet.

3 Es sei darauf hingewiesen, dass hierfür keine weiteren soziodemografischen Variablen kontrolliert wurden. Die Gruppen der Personen mit und ohne Kinder unterscheiden sich hinsichtlich des Alters, was die Ergebnisse verzerren könnte. Im Regressionsmodell, das im Folgenden präsentiert wird, wird für soziodemografische Variablen (inklusive Alter) kontrolliert. Darin wird bestätigt, dass sich Personen mit und ohne Kinder nicht hinsichtlich der Einsamkeit unterscheiden.
